# Combined Age with Mean Decrease Rates of Total Bilirubin and MELD Score as a Novel and Simple Clinical Predictor on 90-Day Transplant-Free Mortality in Adult Patients with Acute Liver Failure Undergoing Plasma Exchange: A Single-Center Retrospective Study

**DOI:** 10.1155/2023/6115499

**Published:** 2023-11-07

**Authors:** Di Jin, Kai Kang, Bing-zhu Yan, Jian-nan Zhang, Jun-bo Zheng, Zhi-hui Wang, Di Wu, Yu-jia Tang, Xin-tong Wang, Qi-qi Lai, Yang Cao, Hong-liang Wang, Yang Gao

**Affiliations:** ^1^Department of Anesthesiology, The Second Affiliated Hospital of Harbin Medical University, Harbin 150086, Heilongjiang Province, China; ^2^Department of Critical Care Medicine, The First Affiliated Hospital of Harbin Medical University, Harbin 150001, Heilongjiang Province, China; ^3^Department of Infectious Diseases, The Second Affiliated Hospital of Harbin Medical University, Harbin 150086, Heilongjiang Province, China; ^4^Department of Critical Care Medicine, The Second Affiliated Hospital of Harbin Medical University, Harbin 150086, Heilongjiang Province, China; ^5^Department of Critical Care Medicine, The Sixth Affiliated Hospital of Harbin Medical University, Harbin 150027, Heilongjiang Province, China; ^6^Institute of Critical Care Medicine, The Sino Russian Medical Research Center of Harbin Medical University, Harbin 150081, Heilongjiang Province, China

## Abstract

**Background:**

Acute liver failure (ALF), previously known as fulminant hepatic failure, has become a common, rapidly progressive, and life-threatening catastrophic hepatic disease in intensive care unit (ICU) due to the continuous increase in drug abuse, viral infection, metabolic insult, and auto-immune cause. At present, plasma exchange (PE) is the main effective alternative treatment for ALF in ICU clinical practice, and high-volume plasma exchange (HVP) has been listed as a grade I recommendation for ALF management in the American Society for Apheresis (ASFA) guidelines. However, no existing models can provide a satisfactory performance for clinical prediction on 90-day transplant-free mortality in adult patients with ALF undergoing PE. Our study aims to identify a novel and simple clinical predictor of 90-day transplant-free mortality in adult patients with ALF undergoing PE.

**Methods:**

This retrospective study contained adult patients with ALF undergoing PE from the Medical ICU (MICU) in the Second Affiliated Hospital of Harbin Medical University between January 2017 and December 2020. Baseline and clinical data were collected and calculated on admission to ICU before PE, including gender, age, height, weight, body mass index (BMI), etiology, total bilirubin, direct bilirubin, indirect bilirubin, prothrombin activity, model for end-stage liver disease (MELD) score, and sequential organ failure assessment (SOFA) score. Enrolled adult patients with ALF undergoing PE were divided into a survival group and a death group at discharge and 90 days on account of medical records and telephone follow-up. After each PE, decreased rates of total bilirubin and MELD score and increased rates of prothrombin activity were calculated according to the clinical parameters. In clinical practice, different patients underwent different times of PE, and thus, mean decrease rates of total bilirubin and MELD score and mean increase rate of prothrombin activity were obtained for further statistical analysis.

**Results:**

A total of 73 adult patients with ALF undergoing 204 PE were included in our retrospective study, and their transplant-free mortality at discharge and 90 days was 6.85% (5/73) and 31.51% (23/73), respectively. All deaths could be attributed to ALF-induced severe and life-threatening complications or even multiple organ dysfunction syndrome (MODS). Most of the enrolled adult patients with ALF were men (76.71%, 56/73), with a median age of 48.77 years. Various hepatitis virus infections, unknown etiology, auto-immune liver disease, drug-induced liver injury, and acute pancreatitis (AP) accounted for 75.34%, 12.33%, 6.85%, 4.11%, and 1.37% of the etiologies in adult patients with ALF, respectively. Univariate analysis showed a significant difference in age, mean decrease rates of total bilirubin and MELD score mean increase rate of prothrombin activity, decrease rates of total bilirubin and MELD score, and increase rate of prothrombin activity after the first PE between the death group and survival group. Multivariate analysis showed that age and mean decrease rates of total bilirubin and MELD score were closely associated with 90-day transplant-free mortality in adult patients with ALF undergoing PE. The 90-day transplant-free mortality was 1.081, 0.908, and 0.893 times of the original value with each one-unit increase in age and mean decrease rates of total bilirubin and MELD score, respectively. The areas under the receiver operatingcharacteristic (ROC) curve of age, mean decrease rates of total bilirubin and MELD score, and the three combined were 0.689, 0.225, 0.123, and 0.912, respectively. The cut-off values of age, mean decrease rates of total bilirubin and MELD score, and the three combined were 61.50, 3.12, 1.21, and 0.33, respectively. The specificity and sensitivity of combined age with mean decrease rates of total bilirubin and MELD score for predicting 90-day transplant-free mortality in adult patients with ALF undergoing PE were 87% and 14%.

**Conclusion:**

Combined age with mean decrease rates of total bilirubin and MELD score as a novel and simple clinical predictor can accurately predict 90-day transplant-free mortality in adult patients with ALF undergoing PE, which is worthy of application and promotion in clinical practice, especially in the identification of potential transplant candidates.

## 1. Introduction

Acute liver failure (ALF), previously known as fulminant hepatic failure, has become a common, rapidly progressive, and life-threatening catastrophic hepatic disease secondary to massive acute hepatocyte necrosis in intensive care unit (ICU) due to the continuous increase in drug abuse, viral infection, metabolic insult, and auto-immune cause. The death and/or dysfunction of massive hepatocyte cells can drive local and systemic inflammatory responses and immune dysfunction in a vicious cycle and eventually lead to a sudden loss of liver function in healthy individuals without preexisting liver disease [1, 2]. The complications of ALF involve almost every organ system, manifested as hepatic encephalopathy (HE), intracranial hypertension, cerebral herniation, hypoglycemia, coagulopathy, hemodynamic instability, renal injury, respiratory failure, irreversible multiple organdysfunction syndrome (MODS), and even death without appropriate and timely intervention or liver transplantation (LT) [3]. In addition, secondary infection is also one of the common complications in patients with ALF due to a deficiency in immune function and an independent predictor of poor outcomes [4]. Therefore, a high-standard cluster infection control strategy should be implemented to minimize the risk of nosocomial infection in the ICU. In clinical practice, the diagnosis of ALF requires a comprehensive and detailed evaluation, including medical history, clinical symptoms, physical examination, laboratory parameters, and imaging findings according to the guidelines for diagnosis and treatment of liver failure from infectious diseases and hepatology branches of the Chinese Medical Association [5]. Prompt recognition, identification of underlying etiology, and effective specific interventions are the cornerstones for adult patients with ALF to obtain a better prognosis.

With the rapid advancement of critical care medicine management and emergency LF (ELT) technology, the outcome of adult patients with ALF has been remarkably improved in the past decade [6, 7]. However, the gap between donor liver availability and the number of potential transplant candidates continues to widen, and thus, donor liver scarcity, economic challenges, and serious posttransplantation complications have become the main obstacles to the clinical application of LT [8–10]. Plasma exchange (PE), especially high-volume plasma exchange (HVP), can offer a survival benefit for ALF patients who are unable or not appropriate for LT [10–15]. At present, PE is the main effective alternative treatment for ALF in ICU clinical practice, and HVP has been listed as a grade I recommendation for ALF management in the American Society for Apheresis (ASFA) guidelines [16, 17]. The mechanisms of PE pertain to the removal of an over-accumulation of various harmful metabolites and toxins to provide a suitable microenvironment, supplement of important physiological substances contained in fresh frozen plasma, modulation of innate and adaptive immune responses to correct imbalanced immunity, promotion of native liver regeneration, and amelioration of multiple organ dysfunction [12, 15, 18–21]. At the same time, due to the strict control and management of collection, transportation, storage, and use of blood products in China, the risk of disease transmission caused by using fresh frozen plasma in the process of PE is very low, but not impossible. Therefore, PE can serve as a safe, well-tolerated, and useful treatment option for patients with ALF and bridging therapy to LT [10, 22].

Prompt and accurate evaluation of the effectiveness of current treatment is of great practical significance for early predicting the prognosis and identifying potential transplant candidates among patients with ALF. To date, none of the related prognostic scores have achieved universal acceptance for clinical prediction on 90-day transplant-free mortality in adult patients with ALF undergoing PE. To address this practical issue, our study aims to identify a novel and simple clinical predictor on 90-day transplant-free mortality in adult patients with ALF undergoing PE by combining baseline data with clinical data from the ICU, which will help intensivist to recognize adult patients with ALF at different risk levels and screen those eligible for LT.

## 2. Materials and Methods

### 2.1. Study Design

This retrospective study contained adult patients with ALF undergoing PE from the Medical ICU (MICU) in the Second Affiliated Hospital of Harbin Medical University between January 2017 and December 2020. Baseline and clinical data were collected and calculated on admission to the ICU before PE. Enrolled adult patients with ALF were divided into a survival group and a death group at discharge and 90 days on account of medical records and telephone follow-up. After each PE, decrease rates of total bilirubin and model for end-stage liver disease (MELD) score and increase rate of prothrombin activity were calculated according to the clinical parameters. In clinical practice, different patients underwent different times of PE, and therefore, mean decrease rates of total bilirubin and MELD score and mean increase rate of prothrombin activity were obtained for further statistical analysis. Information collection and telephone follow-up were carried out by a dedicated intensivist in our research team, and thus, the personal information of the selected patients was strictly confidential. The study protocol was reviewed and approved by the Ethics Committee of the Second Affiliated Hospital of Harbin Medical University (IRB number: KY2021-273). Due to the nature of the retrospective study, the written informed consent of this study was waived.

### 2.2. Study Population

The inclusion criteria of this retrospective study included MICU admission, diagnosis of ALF, undergoing PE, transplantation-free, and older than 18 years of age. The patients who met the following criteria were excluded, including any form of known chronic liver failure (CLF), cirrhosis, hepatic malignancy, obstructive jaundice, previous LT, pregnant or breastfeeding women, and incomplete medical records. All enrolled adult patients with ALF received standard treatment in accordance with the guidelines for diagnosis and treatment of liver failure from the American Gastroenterological Association Institute, the European Association for the Study of Liver Disease, and infectious diseases and hepatology branches of the Chinese Medical Association and were managed by the same group of experienced intensivists in the MICU of the Second Affiliated Hospital of Harbin Medical University [5, 23, 24].

### 2.3. Diagnosis of ALF

A combination of detailed medical history, clinical symptoms and comprehensive physical examination, extensive laboratory parameters (including hepatic and extrahepatic organ function indicators), and imaging findings (for example, abdominal Doppler ultrasound, computed tomography, or magnetic resonance imaging) was necessary for the diagnosis of ALF in accordance with the guideline for diagnosis and treatment of liver failure from infectious diseases and hepatology branches of the Chinese Medical Association [5, 25]. Liver biopsy via a transjugular approach could be considered in some patients with difficult diagnoses [26].

### 2.4. PE Procedure

In clinical practice, the exchange volume of each PE was approximately 3000 ml. The patient's plasma was removed at a rate of 1-2 liters per hour and replaced with an equal volume of fresh frozen plasma through a PE machine (Prismaflex, Gambro Lundia AB, Sweden). Therefore, the duration of PE was usually about 2 hours. 12,500 units of heparin was added into 3000 ml normal saline as preflushing solution for PE, and no additional heparin was administrated anymore during PE due to the poor coagulation function of the enrolled adult patients with ALF. Corticosteroid administration, fluid resuscitation, and vasopressor support could be considered when hypersensitivity or hypotension occurred.

### 2.5. Calculation of Total Bilirubin, Prothrombin Activity, and MELD Score Change Rates

MELD score was calculated as follows: MELD = 3.78 × ln [TBiL (mg/dl)] + 11.2 × ln [INR] + 9.57 × ln [Cr (mg/dl)] + 6.43. Among the formula, TBiL, INR, and Cr, respectively, represented total bilirubin, international standardized ratio, and serum creatinine, while ln, also known as loge, was a natural logarithm. After each PE, routine laboratory parameters were obtained, including total bilirubin, prothrombin activity, international standardized ratio, and serum creatinine, in order to calculate decrease rates of total bilirubin and MELD score and increase rate of prothrombin activity. Mean decrease rates of total bilirubin and MELD score and mean increase rate of prothrombin activity were proposed for further statistical analysis due to different times of PE per selected patient.

### 2.6. Data Collection

Baseline and clinical data, including gender, age, height, weight, body mass index (BMI), etiology, total bilirubin, direct bilirubin, indirect bilirubin, prothrombin activity, MELD score, sequential organ failure assessment (SOFA) score, and hospital day, were collected and calculated from medical records. Enrolled adult patients with ALF underwent different times of PE and then were divided into a survival group and a death group at discharge and 90 day on account of medical records and telephone follow-up. Baseline and clinical data were compared between the two groups in order to assess their predictive values on 90-day transplant-free mortality in adult patients with ALF undergoing PE.

### 2.7. Statistical Analysis

SPSS 22.0 (SPSS Inc., Chicago, IL, United States) was used for statistical analysis. Qualitative data were described as counting, and Chi-square test and Fisher's exact probability method were used for intergroup comparisons, while quantitative data were expressed as mean ± stand deviation (SD), and *t*-test or rank-sum test was employed for intergroup comparisons. Independent sample *t*-test was adopted for the quantitative data conforming to the normal distribution, while Mann–Whitney test was employed for those not conforming to the normal distribution. Multivariate logistic regression was used for multivariate analysis, and receiver operating characteristic (ROC) curves analysis was performed on significant variables to obtain sensitivity, specificity, and cut-off value. A *p* value <0.05 was considered statistically significant.

## 3. Results

### 3.1. Baseline and Clinical Data of Adult Patients with ALF Undergoing PE

A total of 73 adult patients with ALF undergoing 204 PE were included in our retrospective study, and their transplant-free mortality at discharge and 90 days was 6.85% (5/73) and 31.51% (23/73), respectively. All deaths could be attributed to ALF-induced severe and life-threatening complications or even MODS. Most of the enrolled adult patients with ALF were men (76.71%, 56/73), with a median age of 48.77 years. Various hepatitis virus infections, unknown etiology, auto-immune liver disease, drug-induced liver injury, and acute pancreatitis (AP) accounted for 75.34%, 12.33%, 6.85%, 4.11%, and 1.37% of the etiologies in adult patients with ALF, respectively. The remaining baseline and clinical data are shown in Table 1.

### 3.2. Univariate Analysis of 90-Day Transplant-Free Mortality in Adult Patients with ALF Undergoing PE

Univariate analysis showed a significant difference in age, mean decrease rates of total bilirubin and MELD score, mean increase rate of prothrombin activity, decrease rates of total bilirubin and MELD score after the first PE, and increase rate of prothrombin activity after the first PE between the death group and survival group (*p*=0.002, *p* ≤ 0.001, *p* ≤ 0.001, *p*=0.048, *p*=0.003, *p*=0.019, *p*=0.018, respectively) (Table 2).

### 3.3. Multivariate Analysis of 90-Day Transplant-Free Mortality in Adult Patients with ALF Undergoing PE

Multivariate analysis showed that age and mean decrease rates of total bilirubin and MELD score were closely associated with 90-day transplant-free mortality in adult patients with ALF undergoing PE (*p*=0.028, *p*=0.016, *p*=0.015, respectively). The 90-day transplant-free mortality was 1.081, 0.908, and 0.893 times of the original value for each one-unit increase in age and mean decrease rates of total bilirubin and MELD score, respectively (Table 3).

### 3.4. ROC Curve Analysis

**Figure 1:**
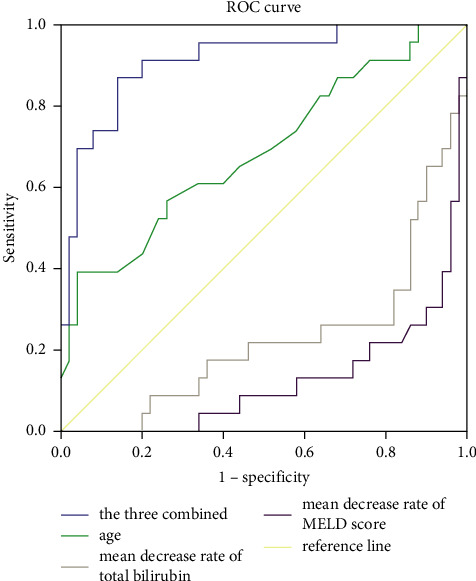
ROC curve analysis. ROC, receiver operating characteristic; MELD score, model for end-stage liver disease.

The areas under the ROC curve of age, mean decrease rates of total bilirubin and MELD score, and the three combined were 0.689, 0.225, 0.123, and 0.912, respectively (Table 4 and Figure 1). The cut-off values of age, mean decrease rates of total bilirubin and MELD score, and the three combined were 61.50, 3.12, 1.21, and 0.33, respectively (Table 5). The specificity and sensitivity of combined age with mean decrease rates of total bilirubin and MELD score for predicting 90-day transplant-free mortality in adult patients with ALF undergoing PE were 87% and 14% (Table 5).

## 4. Discussion

ALF is a severe physiologic dysfunction syndrome and rapid-onset clinical deterioration, with significant morbidity and mortality in previously healthy individuals [27]. Drug toxicity and viral hepatitis constitute the main etiologies of ALF; however, a significant proportion of patients with ALF have no clear underlying etiologies [25, 28]. Significant geographical variation still exists in the etiologies of ALF although it has changed in recent years. For example, viral infection is more prominent in Asia due to a high prevalence of hepatitis B virus (HBV) infection, which is different from other developed countries [29, 30]. Different etiologies of ALF may be one of the determinants of its therapeutic approaches, need for ELT, and even clinical outcomes [31, 32]. ALF usually causes a series of clinical manifestations from decompensation of hepatic and/or extrahepatic organ function and a poor prognosis without LT [28, 33]. At present, ALF still remains a huge clinical challenge with a high transplant-free 90-day mortality as shown in our results. The potential for rapid progression of ALF to MODS is related to activation of innate immune cells and hepatic and systemic inflammatory responses, so it is urgent and important to detect these conditions in time and provide appropriate follow-up interventions [34].

PE is a clinical process of removing plasma from blood in patients and replacing it with fresh frozen plasma. In fact, PE, as an important part of standard treatment for ALF, is one of a variety of blood purification (BP) technologies in clinical application, which can remove harmful and even beneficial mediators without selectivity [35]. As an extracorporeal procedure, it can provide a transient liver function replacement and improve the capacity of the liver to regenerate through a series of protective mechanisms until recovery of the native liver function or LT and thus may be a promising and attractive approach. It has been demonstrated that the clinical parameters of patients with ALF can be improved by PE, which is comparable to the therapeutic effect of HVP with fewer adverse events [15, 36]. Adopting specific standard treatments in the early phase and receiving a good response to them may prevent or delay the progression of ALF to MODS, and be a significant clinical predictor [9]. In the meantime, this method is crucial to early and accurately identify adult patients with ALF who respond poorly to medicine treatment and PE and have minimal capacity for hepatic regeneration, and therefore, they will not survive without LT. It is currently recognized that LT remains the only definitive life-saving ultimate defense for patients with advanced or severely unresolvable ALF although ALF uncommonly results in urgent consideration of LT [25, 37].

Clinically, ALF is mainly manifested as acute onset of jaundice, coagulopathy, and hepatic and/or extra-hepatic organ dysfunction. Total bilirubin level has been confirmed to be positively correlated with the severity of liver injury in patients with acute hepatitis, and hyperbilirubinemia is significantly more frequent and severe in patients with ALF [38, 39]. The phenomenon of “enzyme-jaundice separation” is also a sensitive indicator of liver damage. In addition, prothrombin activity <40% is a common and useful diagnostic marker in patients with ALF [39]. Among HBV-related acute-on-chronic liver failure (ACLF) patients, it has been confirmed that age, levels of total bilirubin, serum creatinine, prothrombin time (PT), and prothrombin activity are independent risk factors of mortality [11, 40, 41]. Arterial lactate concentrations in patients with ALF are not affected by HVP, which neither reduces the lactate production nor interferes with hepatic metabolic clearance [14]. MELD score, calculated only by objective variables, has been validated and widely accepted as an objective and accurate tool to evaluate disease severity and predict prognosis of patients with different advanced liver disease, especially for those with fulminant liver failure, alcoholic hepatitis, cirrhosis, and ACLF, and thus determines organ allocation for LT [3, 42–45]. Certainly, it also has blind zones in prediction. A novel and simple predictor is urgently needed for clinical application to assess disease severity and treatment responsiveness in order to accurately designate adult patients with ALF undergoing PE for appropriate interventions. Therefore, in this study, total bilirubin, prothrombin activity, MELD score, and their change rates affected by PE were collected, calculated, and combined with baseline data to identify a novel and simple clinical predictor on 90-day transplant-free mortality in adult patients with ALF undergoing PE.

In the current study, the 90-day transplant-free mortality was 1.081 times higher for each one-year increase in age over 61.5 years, indicating that age greater than 61.5 was a risk factor for 90-day transplant-free mortality in adult patients with ALF undergoing PE. As for mean decrease rates of total bilirubin and MELD score, the 90-day transplant-free mortality was 0.908 and 0.893 times of the original value for each one-unit increase after greater than 3.12 and 1.21, respectively, suggesting that they were protective factors. Obviously, in clinical practice, the more significant mean decrease rates of total bilirubin and MELD score decreased the better treatment responsiveness of adult patients with ALF to PE and thus the greater possibility of recovery from ALF. The area under the ROC curve of combined age with mean decrease rates of total bilirubin and MELD score was 0.912, which was much larger than that of each variable alone, indicating that the three combined was the best choice to predict 90-day transplant-free mortality in adult patients with ALF undergoing PE, with a specificity of 0.87 and a sensitivity of 0.14. To our knowledge, this is the first time to combine age with mean decrease rates of total bilirubin and MELD score as a novel and simple clinical predictor to predict 90-day transplant-free mortality in adult patients with ALF undergoing PE. Moreover, it is significantly convenient to calculate and apply in the clinical setting. For adult patients with ALF undergoing multiple PE, dynamic assessment of this predictor may predict outcomes better. This novel and simple clinical predictor can be a useful tool to assist intensivists in determining if PE is beneficial for adult patients with ALF and screening potential transplant candidates.

There are several limitations in our study. First, the single-center small-sample retrospective study affects the credibility of our conclusion. Second, the etiologies of ALF are heterogeneous. As mentioned above, different etiologies of ALF may require different regimes and determine the prognosis of adult patients with ALF to a certain extent. Third, the timing of PE intervention is based upon our clinical experience and objective situation and therefore differs between adult patients with ALF. Fourth, combined age with mean decrease rates of total bilirubin and MELD score as a novel and simple clinical predictor is only applicable to adult patients with ALF undergoing PE, not directly extrapolatable to other patient populations, with limited sensitivity. Lastly, our novel clinical predictor needs to be further verified and improved in future well-designed multicenter clinical trials with large samples.

## 5. Conclusion

In summary, the transplant-free mortality of adult patients with ALF undergoing PE at discharge and 90-day was 6.85% and 31.51%, respectively, which meant that nearly a quarter of patients died soon after discharge. Considering the absolute magnitude of the population, there will be a huge patient group. Combined age with mean decrease rates of total bilirubin and MELD score as a novel and simple clinical predictor can accurately predict 90 day transplant-free mortality in adult patients with ALF undergoing PE, which is worthy of application and promotion in clinical practice, especially in the identification of potential transplant candidates.

## Figures and Tables

**Table 1:** Baseline and clinical data of adult patients with ALF undergoing PE.

Variable	*N*	Min	Max	Mean	Std	p25	p50	p75
Age	73	19.00	80.00	48.77	12.85	40.50	48.00	56.00
Height	73	1.52	1.88	1.70	0.09	1.66	1.71	1.76
Weight	73	42.00	110.00	70.67	11.19	62.50	70.00	77.00
BMI	73	14.88	35.84	24.41	3.26	22.45	24.31	26.33
Total bilirubin before PE	73	151.00	850.60	448.35	148.49	354.15	444.90	543.50
Direct bilirubin before PE	73	109.20	686.30	360.44	132.97	257.30	346.00	443.55
Indirect bilirubin before PE	73	1.30	233.80	88.28	52.10	50.35	87.40	117.35
Prothrombin activity before PE	73	13.00	77.00	30.13	12.00	21.00	28.00	35.50
MELD score before PE	73	9.36	48.52	26.67	5.93	22.43	26.85	29.68
SOFA score before PE	73	3.00	8.00	5.10	1.12	4.00	5.00	6.00
Mean decrease rate of total bilirubin	73	−65.51	56.84	10.89	18.11	0.80	12.63	22.04
Mean increase rate of prothrombin activity	73	−48.94	89.36	11.36	19.49	1.75	9.03	18.97
Mean decrease rate of MELD score	73	−48.69	30.37	3.49	13.01	−1.88	5.34	12.52
Decrease rate of total bilirubin after the first PE	73	−65.51	56.84	11.45	21.31	−2.46	14.51	24.59
Increase rate of prothrombin activity after the first PE	73	−48.94	138.10	22.45	32.30	2.19	21.88	40.66
Decrease rate of MELD score after the first PE	73	−48.69	41.35	7.79	15.64	0.73	11.05	19.33
Hospital day	73	3	90	25.95	15.86	15.50	23.00	33.00

ALF, acute liver failure; PE, plasma exchange; BMI, body mass index; MELD score, model for end-stage liver disease; SOFA score, sequential organ failure assessment score.

**Table 2:** Univariate analysis of 90-day transplant-free mortality in adult patients with ALF undergoing PE.

Variable	Death group (*N* = 23)	Survival group (*N* = 50)	*X* ^2^ */t/Z*	*p*
Gender (male/female)	20/3	36/14	1.973	0.160
Age	55.43 ± 13.91	45.70 ± 11.19	−3.194	0.002
Height	1.71 ± 0.07	1.70 ± 0.09	−0.764	0.447
Weight	72.26 ± 11.46	69.94 ± 11.11	−0.821	0.414
BMI	24.54 ± 2.62	24.36 ± 3.53	−0.215	0.830
Total bilirubin before PE	464.53 ± 145.97	440.91 ± 150.51	−0.628	0.532
Direct bilirubin before PE	383.00 ± 137.92	350.06 ± 130.73	−0.983	0.329
Indirect bilirubin before PE	81.52 ± 54.08	91.40 ± 51.42	0.750	0.456
Prothrombin activity before PE	31.58 ± 13.19	29.46 ± 11.49	−0.618	0.537
MELD score before PE	26.55 ± 5.77	26.72 ± 6.07	0.114	0.909
SOFA score before PE	5.43 ± 1.24	4.94 ± 1.04	−1.622	0.105
Mean decrease rate of total bilirubin	−1.22 ± 20.08	16.46 ± 14.17	−3.752	≤0.001
Mean increase rate of prothrombin activity	2.91 ± 27.35	15.25 ± 13.18	2.057	0.048
Mean decrease rate of MELD score	−7.60 ± 14.94	8.59 ± 8.03	4.881	≤0.001
Decrease rate of total bilirubin after the first PE	0.64 ± 24.60	16.42 ± 17.77	3.112	0.003
Increase rate of prothrombin activity after the first PE	7.09 ± 39.10	29.52 ± 26.16	2.505	0.018
Decrease rate of MELD score after the first PE	−0.05 ± 20.68	11.40 ± 11.20	2.494	0.019

ALF, acute liver failure; PE, plasma exchange; BMI, body mass index; MELD score, model for end-stage liver disease; SOFA score, sequential organ failure assessment score.

**Table 3:** Multivariate analysis of 90-day transplant-free mortality in adult patients with ALF undergoing PE.

Variable	B	SE	Wald	DF	*p*	Exp (B)	95% CI for EXP (B)	
							Lower bound	Upper bound
Age	0.078	0.035	4.839	1.000	0.028	1.081	1.009	1.159
Mean decrease rate of total bilirubin	−0.097	0.040	5.756	1.000	0.016	0.908	0.839	0.982
Mean decrease rate of MELD score	−0.113	0.047	5.863	1.000	0.015	0.893	0.815	0.979
Constant	−3.762	1.745	4.647	1.000	0.031	0.023		

ALF, acute liver failure; PE, plasma exchange; SE, standard error; CI, confidence interval; MELD score, model for end-stage liver disease.

**Table 4:** Area under the ROC curve.

Variable	Area	SE	Asymptotic sig.	Asymptotic 95% CI	
				Lower bound	Upper bound
Age	0.689	0.070	0.010	0.552	0.826
Mean decrease rate of total bilirubin	0.225	0.062	0.000	0.104	0.346
Mean decrease rate of MELD score	0.123	0.045	0.000	0.035	0.211
The three combined	0.912	0.037	0.000	0.839	0.985

ROC, receiver operating characteristic; SE, standard error; CI, confidence interval; MELD score, model for end-stage liver disease.

**Table 5:** Cut-off value, and specificity and sensitivity of the ROC curve.

Variable	Cut-off value	Specificity	Sensitivity
Age	61.50	0.39	0.04
Mean decrease rate of total bilirubin	3.12	0.26	0.82
Mean decrease rate of MELD score	1.21	0.22	0.84
The three combined	0.33	0.87	0.14

ROC, receiver operating characteristic; MELD score, model for end-stage liver disease.

## Data Availability

The dataset used and/or analysed during the current study is available from the corresponding author upon reasonable request.
